# Coxiellosis in domestic livestock of Puducherry and Tamil Nadu: Detection of *Coxiella burnetii* DNA by polymerase chain reaction in slaughtered ruminants

**DOI:** 10.14202/vetworld.2017.667-671

**Published:** 2017-06-19

**Authors:** Jothimani Pradeep, Selvaraj Stephen, Pratheesh Pooja, Anbalagan Akshayavardhini, Balakrishnan Sangeetha, Prabakar Xavier Antony

**Affiliations:** 1 Department of Microbiology, Mahatma Gandhi Medical College & Research Institute, Puducherry, India; 2 Department of Genomics and Proteomics, Central Interdisciplinary Research Facility, Mahatma Gandhi Medical College & Research Institute, Puducherry, India; 3 Department of Veterinary Microbiology, Rajiv Gandhi Institute of Veterinary Education and Research, Puducherry, India

**Keywords:** *Coxiella burnetii* DNA, coxiellosis, Trans-polymerase chain reaction

## Abstract

**Background and Aim::**

In the course of our Indian Council of Medical Research project on coxiellosis in Puducherry and Tamil Nadu, 5.64% goat, 1.85% sheep, 1.06% buffaloes, and 0.97% cattle were positive for *Coxiella burnetii* antibodies by enzyme linked immunosorbent assay kit (IDEXX, Liebefeld, Switzerland). In this preliminary study, we have proceeded to look for *C. burnetii* DNA in those antibody positive specimens employing an imported commercial *C. burnetii* polymerase chain reaction (PCR) kit.

**Materials and Methods::**

Blood samples were collected during slaughtering. All 15 blood samples of antibody positive ruminants and three antibody negative samples were subjected to conventional Trans-PCR assay with a commercial PCR kit (Genekam Biotechnology AG, Duisburg, Germany). An in-house Trans-PCR was included in the study for comparison.

**Results::**

A total of 15 antibody positive and three antibody-negative serum samples belonging to 11 goat, 4 sheep, 1 cattle, and 2 buffaloes were tested in duplicate for the presence of *C. burnetii* DNA by the commercial agar gel PCR kit and an in-house Trans-PCR. Only one buffalo serum sample was positive for *C. burnetii* with a band at 243 bp in in-house Trans-PCR.

**Discussion::**

Seropositivity for *C. burnetii* need not necessarily translate into infectivity status of the animal. Conversely, seronegative ruminants can shed *C. burnetii*. Rapid disintegration of *C. burnetii* DNA during the storage period is an important impediment in QF-PCR research. This is the first time the performance of this commercial PCR kit is being validated in India.

**Conclusion::**

Commercial PCR kit, Genekam did not identify any positive sample, probably because it targeted a larger amplicon of 687 bp.

## Introduction

To quote Kovacova and Kazar “Q fever – still a query and underestimated infectious disease” [1]. This disease is prevalent worldwide with the exception of New Zealand [2,3]. *Coxiella burnetii*, causative agent of Q fever is an obligate intracellular Gram-negative bacterium. It is a potential agent of bioterrorism and Category-B pathogen demanding bio-safety level-3 facilities for isolation/antigen preparation works [4-8]. Hence, only serological and molecular diagnostic tests are available to most of the laboratories in the world. Coxiellosis is a major zoonotic disease and it infects a wide spectrum of animals such as ruminants, dogs, cats, reptiles, wild animals, and birds [2,7-9]. Commonly the dog ticks (*Rhipicephalus sanguineus*) and occasionally snake ticks (*Aponomma gervaisi*) do harbor *C. burnetii* [10,11] and may transmit infection to the animals but normally have no role to play in human illness. Interestingly, a report mentions that the crushing of infected tick between the fingers has resulted in Q fever [12]. Transmission of Q fever from farm animals is an important reservoir of human infections. It can transmit through inhalation/ingestion of aerosols by infected aborted materials, unpasteurized milk and its products [8,13,14]. In India, the first two cases of human Q fever were reported by Anderson and Kalra in 1954 [15] and Ghosh and Rao in 1956 [16], followed by countrywide serological surveys by Kalra and Taneja [17]. Two reviews of Q fever in man and animals of India appeared in 1978 and 1980, giving detailed account of seroprevalence as well as tests employed by earlier workers [18,19]. The late seventies and early eighties witnessed several reports of Q fever in human/animals from several states such as Punjab, Haryana, Rajasthan, Kerala, Karnataka, Uttar Pradesh, Maharashtra, Delhi, Orissa, and the latest from Rajasthan (2003), Tamil Nadu (2008), and Puducherry (2014) [20-32]. No serosurvey reports on coxiellosis in Indian animals appeared after the eighties, until the recent report of coxiellosis in small ruminants of Puducherry in 2014 [32]. Evidence of animal and human abortions, neonatal septicemia, endocarditis, and atypical pneumonia due to *C. burnetii* based on immunofluroescence test/polymerase chain reaction (PCR) are recorded in recent Indian literature [4,5,33,34].

In the recent times, coxiellosis in animals have been reported from several countries such as Bangladesh, Iran, Brazil, Turkey, USA, Greece, Bulgaria, Switzerland, Italy, and The Netherlands [14,35,36]. An outbreak of Q fever in Danish goat, leading to killing of 51,680 infected goats and reports of coxiellosis in different countries across the globe have raised the awareness level of Q fever throughout the world [36]. Nearly, 583 abortions had occurred due to *C. burnetii* infection in small ruminants between 2002 and 2011. An observation of *C. burnetii* infection in Swiss animals by screening of milk samples shows that mostly it occurred in <5% of cattle products and absent in sheep or goat samples [37].

Seroprevalence studies of coxiellosis in several countries including India were based on specific and sensitive serological tests such as capillary agglutination test, complement fixation test, enzyme-linked immunosorbent assay (ELISA), indirect immunofluroescence assay, molecular tests like PCR, real-time PCR, and loop-mediated isothermal amplification [4-43]. Our aim of this preliminary communication is to examine a small number of seropositive ruminants for *C. burnetii* DNA. Evaluation of a commercial and imported conventional PCR kit is done for the first time in India and compared with an in-house prepared Trans-PCR.

## Materials and Methods

### Ethical approval

Institute’s Animal Ethical Committee had given approval for this work.

### Study area

This study was conducted in the Microbiology Department of a tertiary care super specialty teaching hospital at Puducherry during January 2014 to December 2015.

### Collection of blood samples

Blood samples were collected from domestic livestock at the time of slaughtering from various private/government/municipal abattoirs as well as mutton shops located in different areas of Tamil Nadu and Puducherry. A total of 772 blood samples were collected comprising 216 sheep, 195 goat, 206 cattle, and 188 buffaloes.

### Test procedure for ELISA and PCR

ELISA was performed with Q fever antibody ELISA Test Kit, (IDEXX, Liebefeld, Switzerland). The ELISA wells were coated with *C. burnetii* Phase I and II antigens and it was carried out as per the procedure outlined by the kit manufacturer and as per earlier report [6].

### DNA extraction

About 200 µl serum samples were used for the genomic DNA extractions as per the manufacturer’s protocol. Carrier DNA was added to the serum sample before extraction to maximize DNA yield, as recommended by the protocol. The purity of the extracted DNA was determined by calculating absorbance (A) A260/A280 ratio, which was in the range of 1.7-1.8 for all the samples. The samples were aliquoted and stored at −80°C till further use.

### Kit-based Trans-PCR

Trans-PCR for the samples was done in duplicates using *C. burnetii* PCR kit (Genekam, Disburg, Germany) as per manufacturer’s protocol. The reaction conditions for the PCR were as follows: Denaturation at 20 s at 95°C, annealing for 60 s at 50°C, and extension for 120 s at 72°C for 30 cycles. The PCR was carried out using C1000 Thermocycler, Bio-Rad, USA, and the PCR product was visualized on agarose gel by ethidium bromide staining.

### In-house Trans-PCR

In-house PCR was carried out using primers (Sigma-Aldrich, Bengaluru) for amplifying IS1111 transposon–like repetitive element of *C. burnetii* [2,9,10]. The sequence of the primers used was Trans 1: 5’-TATGTATCCACCGTAGCCAGTC-3’ and Trans 2: 5’-CCCAACAACACCTCCTTATTC-3’; Trans 3: 5’-GTAACGATGCGCAGGCGAT-3’ and Trans 4: 5’-CCACCGCTTCGCTCGCTA-3’. The PCR reaction was carried out in 25 µl of reaction mixture with 12.5 µl ×2 Taq DNA polymerase PCR kit (ampliqon), 1 µl of forward primer and reverse primer each, 5 µl template DNA, and 5.5 µl molecular grade distilled water. The PCR conditions were as follows:

Denaturation at 95°C for 30 s, annealing at 65°C for 40 s extension at 72°C for 30 s for 35 cycles, and a final elongation step at 72°C for 5 min. The PCR was carried out using C1000 Thermocycler, Bio-Rad, USA and the PCR product was visualized on agarose gel by ethidium bromide staining.

## Results and Discussion

**Figure-1:**
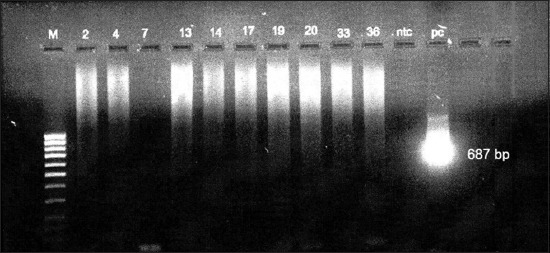
*Coxiella burnetii* Genekam conventional polymerase chain reaction. m – DNA ladder 100 bp; 2, 4, 7, 13, 14, 17, 19, 20, 33, 36 – Negative; ntc – negative control; pc – positive control (687 bp).

**Figure-2:**
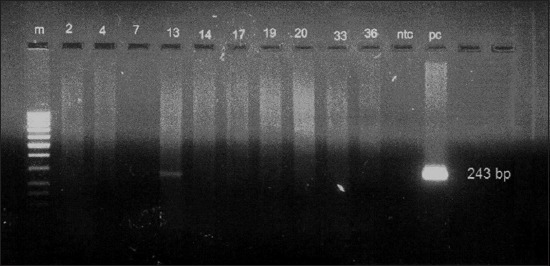
*Coxiella burnetii* in-house Trans-polymerase chain reaction (Trans 3 and 4 primers). m – DNA ladder 100 bp; 2, 4, 7, 14, 17, 19, 20, 33, 36 – Negative; 13 – Positive for *C. brunetti* DNA; ntc – negative control; pc – positive control (243 bp).

In this study, we used four primers Trans 1, 2, 3, and 4 which target IS1111 transposon-gene repetitive element for detection of *C. burnetii* in serum samples. 18 samples, 15 antibody positive, and three antibody-negative serum samples belonging to 11 goat, 4 sheep, 1 cattle, and 2 buffaloes were tested with ready to use conventional Genekam PCR kit with the primer set of Trans 1 and 2, with an expected amplicon size of 687 bp, but none of them were positive. The positive control provided in the kit, as well as *C. burnetii* DNA (Bioscience, Bratislava) gave clear band at 687 bp (Figure-1). Negative controls were added in each run to avoid DNA contamination. When our in-house Trans-PCR was performed with primer set of Trans 1 and 2, all samples were negative except the positive controls. However, when the experiment was repeated with the second set of primers, viz., Trans 3 and 4, one seropositive buffalo had *C. burnetii* DNA (Figure-2).

In this study, we evaluated commercially available, ready to use *C. burnetii* conventional PCR for the first time in India and found it unsatisfactory for Indian ruminants, which is most likely because it targets a larger molecular weight amplicon size 687 bp. Q fever is on the increase and included in the list of notifiable diseases in some European countries [36,39].

Largest outbreak in Bulgaria and Europe lead to the investigators to find out a high seroprevalence of *C. burnetii* antibodies in humans as well as animals [36,42]. According to Bellini *et al.*, only 14% *C. burnetii* antibody positive (by ELISA) ruminants had *C. burnetii* DNA in the real time PCR assay [44]. However, Kargar *et al*. reported that the primers Trans 1 and 2 are highly sensitive and showed 17.14% positivity when compared with Com1 and Coc-PCR with only 10% positivity for milk products [9]. However, Kılıç *et al*. reported that Trans 1 and 2 PCR could detect only 2% *C. burnetii* DNA in the organs of infected animals and observed that IS1111 gene was circulating among the domestic sheep and goat [40]. Gunaydin *et* *al*. reported that Trans PCR 687 bp displayed a negative result in animal serum samples which are seropositive for *C. burnetii* antibodies [45]. According to a recent report by Chakarabarthy *et al.*, the overall seroprevalence of coxiellosis in Bangladesh was 7.6% and 6.1% for goats and cattle, respectively. However, none of seropositive samples were positive for *C. burnetii* DNA in real-time PCR [35]. Two researchers observed that *C. burnetii* appears to be quite frequent in blood as well as milk products of cattle [14,35]. Kim *et al*. accounted year wise prevalence of coxiellosis in milk products of cattle as 52.8%, 23.5%, and 31.3% in 2002, 2003, and 2004, respectively [14].

There is not much of a progress in India in the molecular diagnosis of coxiellosis/Q fever, except for few reports from Uttar Pradesh, Karnataka, and Tamil Nadu [4-7,31,34]. By the application of molecular diagnosis using Trans-PCR, Indian researchers reported prevalence of Q fever 21.6% of human abortions, 11.05% of domestic animals with reproductive disorders, and 2.8% of patients with atypical pneumonia [4,5,34]. Our study points to the fact that Trans 3 and 4 are sensitive than Trans 1 and 2, as it is a short fragment in the most conserved region of *C. burnetii* DNA. Use of 243 bp or even smaller 70 bp amplicon [34] could perhaps pick up more positive cases in humans as well as domestic livestock. Das *et al*. isolated *C. burnetii* from the aborted fetuses of 4.54% cattle and 8.33% buffaloes [6]. IS1111 Transposon-repetitive element is best known target for detection of *C. burnetii* DNA in patients with active infections [6,40]. Degradation of preserved *C. burnetii* DNA is a major drawback in the molecular diagnosis of coxiellosis. It is known that while *C. burnetii* antibody positive animals need not necessarily shed this organism, antibody negative livestock can shed this bacterium in their secretions/excretions [32]. In our preliminary work, only a small number of 18 ruminants’ serum samples were used for detection of *C. burnetii* DNA. The study could be expanded to cover more number of animals so as to get a better and clear picture of the prevalence of this zoonosis.

## Conclusion

This preliminary communication records a low (6.67%) *C. burnetii* DNA among antibody positive ruminants. The use of smaller amplicon size of 243 bp and perhaps even much smaller ones might result in a higher percentage of positivity. The large sized amplicon of 687 bp is perhaps a reason for the failure of the commercial kit to detect any positive case.

## Authors’ Contributions

SS, JP, PXA planned, designed, and conducted data interpretation. BS and JP performed sample collection. BS, JP, AA carried out sample analysis. PXA and PP edited the manuscript. All authors read and approved the final manuscript.

## References

[1] Kovácová E, Kazár J (2002). Rickettsial diseases and their serological diagnosis. Acta Virol.

[2] World Organization for Animal Health (2013). Manual of Diagnostic Tests and Vaccines for Terrestrial Animals.

[3] Hilbink F, Penrose M, Kovacova E, Kazar J (1993). Q fever is absent from New Zealand. Int. J. Epidemiol.

[4] Vaidya V.M, Malik S.V.S, Bhilegaonkar K.N, Rathore R.S, Kaur S, Barbuddhe S.B (2010). Prevalence of Q fever in domestic animals with reproductive disorders. Comp. Immunol. Microbiol. Infect. Dis.

[5] Vaidya V.M, Malik S.V.S, Kaur S, Kumar S, Barbuddhe S.B (2008). Comparison of PCR, IF, pathology and isolation for Q fever in humans with spontaneous abortion. J. Clin. Microbiol.

[6] Das D.P, Malik S.V.S, Rawool D.B, Das S, Shoukat S, Gandham R.K, Saxena S, Singh R, Doijad S.P, Barbuddhe S.B (2014). Isolation of *Coxiella burnetii* from bovines with history of reproductive disorders in India and phylogenetic inference based on the partial sequencing of IS1111 element. Infect. Genet. Evol.

[7] Das D.P, Malik S.V.S, Mohan V, Rawool D.B, Barbudhe S.B (2013). Screening of fecal droppings of wild birds for coxiellosis by a duplex PCR targeting Com1 and IS1111 genes of *Coxiella burnetii*. J. Food Borne Zoonotic Dis.

[8] Prasad B.N, Chandiramani N.K, Wagle A (1986). Isolation of *Coxiella burnetii* from human sources. Int. J. Zoonoses.

[9] Kargar M, Rashidi A, Doosti A, Najafi A, Ghorbani-Dalini S (2015). The sensitivity of the PCR method for detection of *Coxiella burnetii* in the milk samples. Zahedan J. Res. Med. Sci.

[10] Stephen S, Chandrashekara I, Rao K.N (1980). Natural occurrence of *Coxiella burnetii* in the brown dog tick *Rhipicephalus sanguineus*. Indian J. Med. Res.

[11] Stephen S, Rao K.N.A (1979). Q fever in South Kanara district:Natural occurrence of *Coxiella burnetii* in the tick (*Aponomma gervaisi*)--preliminary report. Indian J. Med. Res.

[12] Mediannikov O, Fenollar F, Socolovschi C, Diatta G, Bassene H, Molez J.F, Sokhna C, Trape J.F, Raoult D (2010). *C. burnetii* in humans and ticks in rural sengal. PLoS. Negl. Trop. Dis.

[13] Muskens J, Engelen E.V, Maanen C.V, Bartels C, Lam T.J.G (2011). Prevalence of *Coxiella burnetii* infection in Dutch dairy herds based on testing bulk tank milk and individual samples by PCR and ELISA. Vet. Rec.

[14] Kim S.G, Kim E.H, Lafferty C.J, Dubovi E (2005). *Coxiella burnetii* in bulk tank milk samples, United States. Emerg. Infect. Dis.

[15] Anderson P.K, Kalra S.L (1954). Q fever studies in India:A case of human Q fever. Indian J. Med. Res.

[16] Ghosh B, Rao K.N.A (1956). ’Q’fever - A case report. AF. Med. J. India.

[17] Kalra S.L, Taneja B.L (1954). Q fever in India:A serological survey. Indian J. Med. Res.

[18] Padbidri V.S, Gupta N.P (1978). Riskettsiosis in India:A review. J. Indian Med. Assoc.

[19] Stephen S, Rao K.N.A (1980). Q fever in India:A review. J. Indian Med. Assoc.

[20] Randhawa A.S, Dhillon S.S, Jolley W.B (1973). Serologic prevalence of Q fever in the state of Punjab, India. Am. J. Epidemiol.

[21] Sodhi S.S, Joshi D.V, Sharma D.R, Baxi K.K (1980). Seroprevalence of brucellosis and Q fever in dairy animals. Zentralbl. Veterinarmed. B.

[22] Joshi M.V, Padbidri V.S, Rodrigues F.M, Gupta N.P (1979). Prevalence of *Coxiella burnetii* infection among humans and domestic animals of Rajasthan State, India. J. Hyg. Epidemiol. Microbiol. Immunol.

[23] Shanmugam J, Raveendranath M, Sukumaran M (1978). Seroprevalence of Q fever infection in human beings from southern region of Kerala State. Indian J. Med. Res.

[24] Joshi M.V, Menon R.D, Padbidri V.S, Manjrekar S.L (1975). A note on the serological evidence of ’Q’fever in sheep from Karnataka State. Indian J. Anim. Sci.

[25] Stephen S, Indrani R, Rao K.N.A (1978). Q fever antibodies in domestic animals in South Kanara - A preliminary report. Indian J. Med. Res.

[26] Stephen S, Rao K.N (1979). Coxiellosis in reptiles of South Kanara district, Karnataka. Indian J Med Res.

[27] Stephen S, Chandrashekara I, Rao H.L, Rao K.G, Rao K.N (1980). Prevalence of human Q fever in South Kanara district, Karnataka. Indian J. Med. Res.

[28] Yadav M.P, Sethi M.S (1979). Sero-epidemiological studies on coxiellosis in animals and man in the state of Uttar Pradesh and Delhi (India). Int. J. Zoonoses.

[29] Padbidri V.S, Rodrigues F.M, Vidyasagar J, Joshi M.V (1981). Prevalence of antibodies to *Coxiella burnetii* among the domestic animal population in Orissa. Indian J. Med. Vet.

[30] Rao R (2003). Seroprevalence of human coxiellosis Q-fever in Bikaner district of Rajasthan. J. Vet. Public Health.

[31] Balakrishnan N, Menon T, Fournier P.E, Raoult D (2008). *Bartonella quintana* and *Coxiella burnetii* as causes of endocarditis, India. Emerg. Infect. Dis.

[32] Stephen S, Sangeetha B, Antony P.X (2014). Seroprevalence of coxiellosis (Q fever) in sheep and goat in Puducherry and neighbouring Tamil Nadu. Indian J. Med. Res.

[33] Aarthi P, Bagyalakshmi R, Mohan K.R, Krishna M, Nitin M, Madhavan H.N, Kalyani S (2013). First case series of emerging rickettsial neonatal sepsis identified by polymerase chain reaction-based deoxyribonucleic acid sequencing. Indian J. Med. Microbiol.

[34] Gangoliya S.R, Kumar S, Alam S.I, Devi D.R.G, Guchhait P (2016). First molecular evidence of *Coxiella burnetii* in patients with atypical pneumonia, India. J. Med. Microbiol.

[35] Chakarabarthy A, Bhattacharjee P.K, Sarker R.R, Rahman A.K.M, Henning K, Neubauer H, Rahman M.S (2016). Prevalence of *Coxiella burnetii* infection in cattle, black bengal goats and ticks in Bangladesh. Bangladesh J. Vet. Med.

[36] van der Hoek W, Dijkstra F, Schimmer B, Schneeberger P.M, Vellema P, Wijkmans C, Ter Schegget R, Hackert V, van Duynhoven Y (2010). Q fever in the Netherlands:An update on the epidemiology and control measures. Eur. Surveill.

[37] Fretz R, Schaeren W, Tanner M, Baumgartner A (2007). Screening of various foodstuffs for occurrence of *Coxiella burnetii* in Switzerland. Int. J. Food Microbiol.

[38] Edalati-Shokat H, Abbasi-Doulatshahi E, Hajian-Bidar H, Gharekhani J, Rezaei A.A (2015). Q fever in domestic ruminants:A seroepidemiological survey in hamedan, Iran. Int. J. Curr. Microbiol. Appl. Sci.

[39] Mares-Guia M.A, Rozental T, Guterres A, Gomes R, Almeida D.N, Moreira N.S, Barreira J.D, Favacho A.R, Santana A.L, Lemos E.R (2014). Molecular identification of the agent of Q fever-*Coxiella burnetii*-in domestic animals in State of Rio de Janeiro, Brazil. Rev. Soc. Bras. Med. Trop.

[40] Kılıç A, Kalender H, Koç O, Kılınç Ü, Irehan B, Berri M (2016). Molecular investigation of *Coxiella burnetii* infections in aborted sheep in eastern Turkey. Iran. J. Vet. Res.

[41] Bisias G, Burriel A.R, Boutsini S, Kritas S.K, Leontides L.S (2010). A serological investigation of some abortion causes among small ruminant flocks in Greece. Internet J. Vet. Med.

[42] Panaiotov S, Ciccozzi M, Brankova N, Levterova V, Mitova-Tiholova M, Amicosante M, Rezza G, Kantardjiev T (2009). An outbreak of Q fever in Bulgaria. Ann. Ist. Super. Sanita.

[43] Raele D.A, Garofolo G, Galante D, Cafiero M.A (2015). Molecular detection of *Coxiella burnetii* using an alternative Loop-mediated isothermal amplification assay (LAMP). Vet. Ital.

[44] Bellini C, Magouras I, Chapuis-Taillard C, Clerc O, Masserey E, Peduto G, Peter O, Schaerrer S, Schuepbach G, Greub G (2014). Q fever outbreak in the terraced vineyards of Lavaux, Switzerland. N. Microbe. N. Infect.

[45] Gunaydin E, Pekkaya S, Mustak H.K, Dalkilic B (2014). Investigation of Q fever in Kilis and Shamil goats by ELISA and touchdown-PCR. Ankara Univ. Vet. Fak. Derg.

